# Goal-dependent tuning of muscle spindle receptors during movement preparation

**DOI:** 10.1126/sciadv.abe0401

**Published:** 2021-02-24

**Authors:** Stylianos Papaioannou, Michael Dimitriou

**Affiliations:** Physiology Section, Department of Integrative Medical Biology, University of Umeå, S-901 87 Umeå, Sweden.

## Abstract

Voluntary movements are believed to undergo preparation before they are executed. Preparatory activity can benefit reaction time and the quality of planned movements, but the neural mechanisms at work during preparation are unclear. For example, there are no overt changes in muscle force during preparation. Here, using an instructed-delay manual task, we demonstrate a decrease in human muscle afferent activity (primary spindles) when preparing to reach targets in directions associated with stretch of the spindle-bearing muscle. This goal-dependent modulation of proprioceptors began early after target onset but was markedly stronger at the latter parts of the preparatory period. Moreover, whole-arm perturbations during reach preparation revealed a modulation of stretch reflex gains (shoulder and upper arm muscles) that reflected the observed changes in spindle activity. We suggest that one function of central preparatory activity is to tune muscle stiffness according to task goals via the independent control of muscle spindle sensors.

## Teaser

Movement preparation tunes muscle stiffness via the goal-dependent control of muscle spindle sensors.

## INTRODUCTION

A key mission in sensorimotor neuroscience is to understand the function and consequence of “preparatory activity”: the vigorous changes in neural activity that occur in multiple areas of the brain before a planned voluntary movement (*1*–*3*). Although the firing of such “preparatory” neurons has been linked to a variety of factors such as movement direction/extent (*4*, *5*) and visual target location (*6*), the specific function of preparatory activity has remained unclear. A previous claim that preparatory activity represents a subthreshold version of movement-related cortical activity (*2*) has been contradicted more recently in support of the notion that preparation sets another initial dynamical state that promotes execution of the planned movement (*7*). However, it is unclear what this initial state actually entails and by which neural mechanisms exactly the benefits of movement preparation are realized. For example, preparation benefits performance by lowering reaction time (*8*), with longer preparation delays generally leading to better movement quality (*9*), but there are no overt changes in skeletal muscle activity during movement preparation. Moreover, recent behavioral findings indicate that preparation is mechanistically independent from movement initiation, with a distinct neural basis (*10*).

Little attention has been placed on the possibility that preparatory activity may also reflect control of sensory (i.e., proprioceptive) elements located in the peripheral nervous system. The aim of the current study was to investigate the impact of goal-directed movement preparation on muscle spindle firing and assess any implications for “reflex” motor responses. Independent modulation of spindle sensitivity/gain to dynamic muscle stretch (via the γ motor or “fusimotor” system) could function as movement-related preparation that does not determine concurrent skeletal muscle activity but can nevertheless affect the execution of movement through influencing stretch reflex responses of all latencies, i.e., both short-latency reflex (SLR) responses engaging spinal circuits and long-latency reflex (LLR) responses involving supraspinal centers. In other words, we hypothesize that preparatory activity in the brain may underlie goal-dependent changes in muscle stiffness by selectively modulating spindle output, i.e., the negative feedback to the muscle motor drive, which, in turn, affects the mechanical compliance of the muscle to stretch.

In what follows, we describe positive findings generated by three independent but complementary experiments, each using a different group of human participants. One experiment focused on recording spindle afferent activity from hand- and digit-actuating muscles using microneurography (experiment “1”). The other two experiments used a robotic manipulandum platform to study reflex motor responses at the level of the whole arm (experiments “2” and “3”). To our knowledge, experiment 1 represents the first instance where muscle afferent activity was recorded in a context involving both a dedicated movement preparation period and active reaching. Recording from single spindle afferents rather than single fusimotor efferents is not only feasible but also preferable in our paradigm involving active humans. That is, the result of any substantial change in γ activity is a change in the responses of the muscle spindle, and the spindle organ acts as an integrator of input from multiple fusimotor fibers (*11*).

## RESULTS

### Muscle afferent signals in delayed reaching

**Fig. 1:**
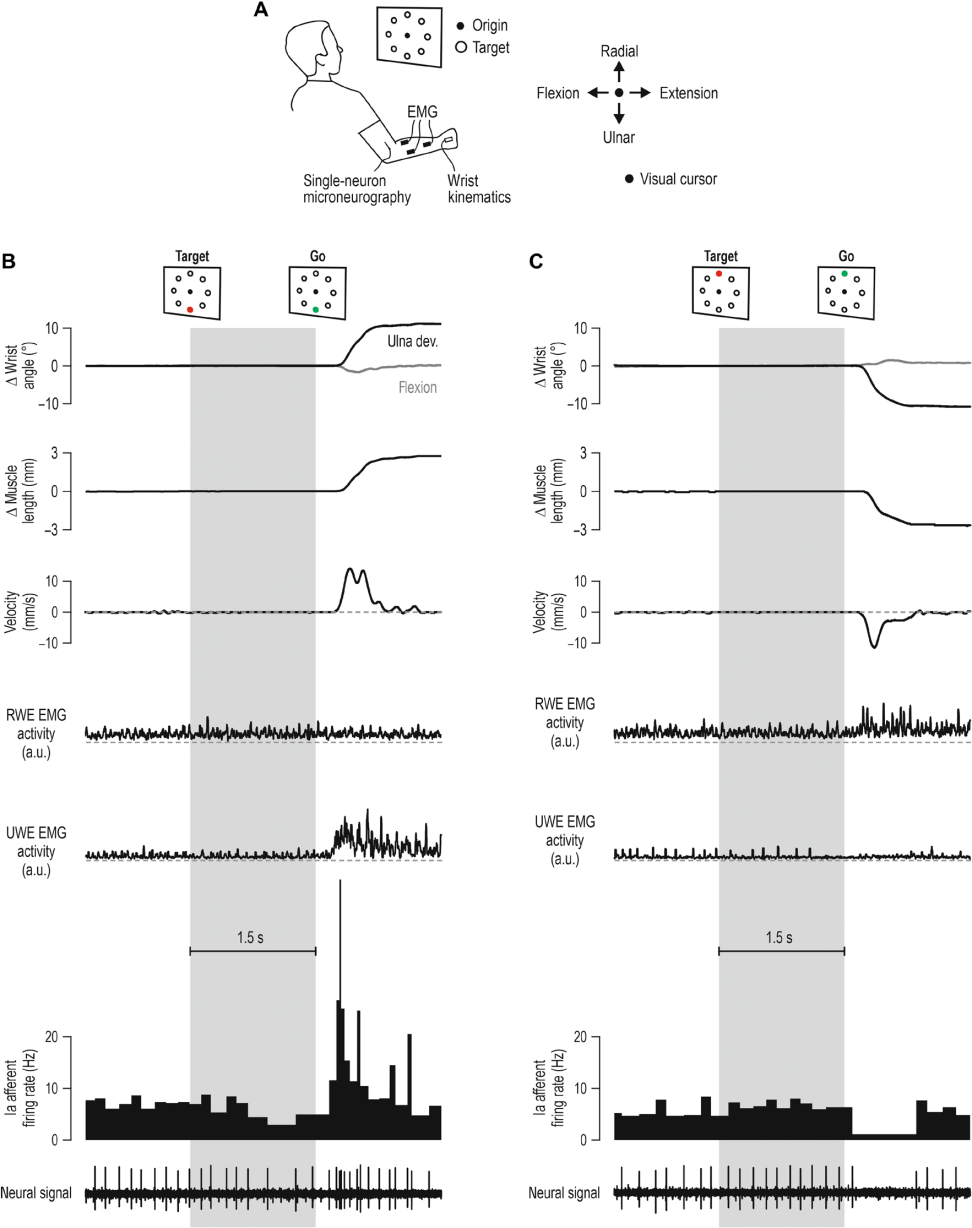
First experimental setup and representative single-trial data. (**A**) The general setup of experiment 1. Participants performed the classic instructed-delay reaching task using their right hand. From an initial semipronated position, wrist flexion-extension moved a visual cursor in the horizontal dimension, and wrist ulna-radial deviation moved the cursor in the vertical dimension. The participant’s task was to move the cursor to reach one of eight peripheral visual targets. On each trial, a target would suddenly turn into a red filled circle, representing the target “cue,” and participants were instructed to move to this target as soon as the go cue appeared (target turned into a green outline). The targets/trials were presented in a block-randomized manner; hence, there was no systematic difference in movement history across a particular group of targets. (**B**) Representative data from a single trial where reaching the target required ulna deviation of the wrist. Muscle length and velocity estimates pertain to the spindle-bearing muscle, which in this case is the radial wrist extensor (RWE; i.e., extensor carpi radialis). Also shown is surface EMG from the ulna wrist extensor muscle (UWE; i.e., extensor carpi ulnaris), which mostly powered the reaching movement. Despite no overt changes in kinematics or EMG during the preparatory period (gray background), primary spindle afferent (Ia) firing rate decreased, particularly at the latter half of this period. (**C**) The same neuron as in (B), but here, the visual target was in the opposite direction, requiring radial deviation at the wrist and therefore shortening of the radial wrist extensor. No decrease in firing rate was observed during the preparatory period. Throughout, dashed gray lines represent zero values. a.u., arbitrary units.

**Fig. 2:**
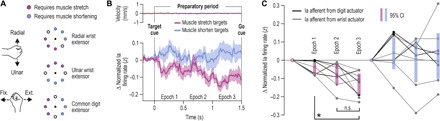
Goal-dependent tuning of muscle spindle receptors during movement preparation. (**A**) The visual targets were categorized on the basis of whether reaching them required stretching or shortening of the spindle-bearing muscle. According to published physiological models for each muscle (see Materials and Methods), six targets represented clear and substantial change in muscle length, whereas two “intermediate” targets (circle outlines) represented little or no muscle stretch or shortening. (**B**) Top: Mean stretch velocity of the recorded spindle-bearing muscles, essentially indicating that no overt movement occurred in the preparatory period (see, e.g., velocity scales in Fig. 1, B and C, and fig. S1). Bottom: Mean change in primary spindle afferent (Ia) firing rates (eight afferents recorded from six individuals). The traces are aligned to onset of the target cue (time “0”). Purple and blue traces represent targets associated with stretch and shortening of the spindle-bearing muscle, respectively. Shading represents ±1 SEM. (**C**) Average Ia firing rates in the three epochs (1 to 3) as shown in (B). Thin gray lines represent individual Ia afferents from wrist extensor muscles, and thin black lines represent Ia afferents from digit extensors. The shaded bars represent 95% CIs, and asterisk represents *P* < 0.05 following a paired *t* test. The same color scheme is used throughout. In the absence of changes in the muscle’s mechanical state, goal-dependent decreases in tonic Ia firing rate indicate a goal-dependent change in the fusimotor drive to spindles; such fusimotor supply may possibly have a stronger effect on the spindles’ sensitivity to dynamic muscle stretch (i.e., gain). n.s., not significant.

In experiment 1, participants performed the classic instructed-delay reaching task with the right hand while we simultaneously recorded hand kinematics, relevant electromyography (EMG) signals, and single afferent activity from wrist or digit extensor muscles (Fig. 1A). Figure 1 (B and C) presents exemplary single-trial data pertaining to the same primary spindle afferent (type “Ia” afferent). Despite no overt changes in kinematic variables or EMG during movement preparation, i.e., during the instructed delay, there was a decrease in the afferent’s firing rate when preparing to reach a target that required stretch of the spindle-bearing muscle (Fig. 1B). However, no such decrease occurred when preparing to move in the opposite direction that required shortening the muscle (Fig. 1C). From each participant in experiment 1, we recorded muscle afferent activity from one of three muscles: the radial wrist extensor (“extensor carpi radialis”), the ulna wrist extensor (extensor carpi ulnaris), or the common digit extensor (extensor digitorum communis). Single trials were categorized according to whether reaching the cued target required a substantial stretch or shortening of the spindle-bearing muscle (Fig. 2A). Despite no overt movement during the preparatory period (Fig. 2B, top), type Ia population responses decreased when preparing to reach targets associated with stretch of the spindle-bearing muscle, relative to baseline (the latter is defined as values in the 0.5-s epoch before target cue onset). The relative suppression effect appeared ~80 ms after onset of the target cue and generally seemed to intensify closer to the onset of the “go” cue (Fig. 2B). Single-sample *t* tests confirmed the range of CIs plotted in Fig. 2C. Type Ia firing rates in all three epochs pertaining to subsequent muscle stretch (purple) were significantly different from baseline [epoch “1”: *t*(7) = −3.3 and *P* = 0.013; epoch “2”: *t*(7) = −3.1 and *P* = 0.017; epoch “3”: *t*(7) = −5.4 and *P* = 0.001], but this was not the case for targets associated with subsequent muscle shortening (“blue”; all *P* > 0.33). A repeated-measures analysis of variance (ANOVA) of the design 2 (target direction) × 3 (epoch) showed a main effect of target direction on Ia firing rates (“stretch” < “shortening” targets) with *F*(1, 7) = 10.8, *P* = 0.013, and η_p_^2^ = 0.6, but the main effect and interaction involving the “epoch” condition were not significant (*P* > 0.1). However, complementary planned comparison tests indicated that firing rates were lower in epoch 3 versus epoch 1 when preparing stretch (purple), with *F*(1, 7) = 10.5 and *P* = 0.014, but there was no significant difference in firing rate between epochs 2 and 3 (*P* = 0.08).

Kinematic and surface EMG signals showed no systematic variation in the preparatory period as a function of target cue (figs. S1 and S2). *T* tests indicated no significant deviations from baseline during preparation for spindle-bearing muscle length (all *P* > 0.36), velocity (all *P* > 0.28), acceleration (all *P* > 0.19), or EMG (all *P* > 0.14), and no variable showed a trend or tendency toward the suppression pattern seen in spindle Ia responses before muscle stretch. We also recorded from four secondary spindle afferents (type “II”) and three Golgi tendon organ afferents (type “Ib” encoding muscle-tendon tension) during the delayed-reach task. The same *t* test analyses as above indicated no difference from baseline in type II firing rates (all *P* > 0.36; fig. S3A) and no tendency toward the suppression pattern seen in spindle Ia responses. There also seemed to be an increase in type Ib firing rates regardless of target group (fig. S3B), and this did not parallel the state of the relevant parent EMG (fig. S3C). Although single-sample *t* tests showed no difference from baseline in type Ib responses (all *P* > 0.09), a 2 (target group) × 3 (epoch) repeated-measures ANOVA yielded a main effect of target group [*F*(1, 2) = 21, *P* = 0.044, and η_p_^2^ = 0.9], indicating that the increases in Ib firing rate were larger for shortening targets. There was no significant effect of epoch or interaction effect between target group and epoch (*P* > 0.21).

**Fig. 3:**
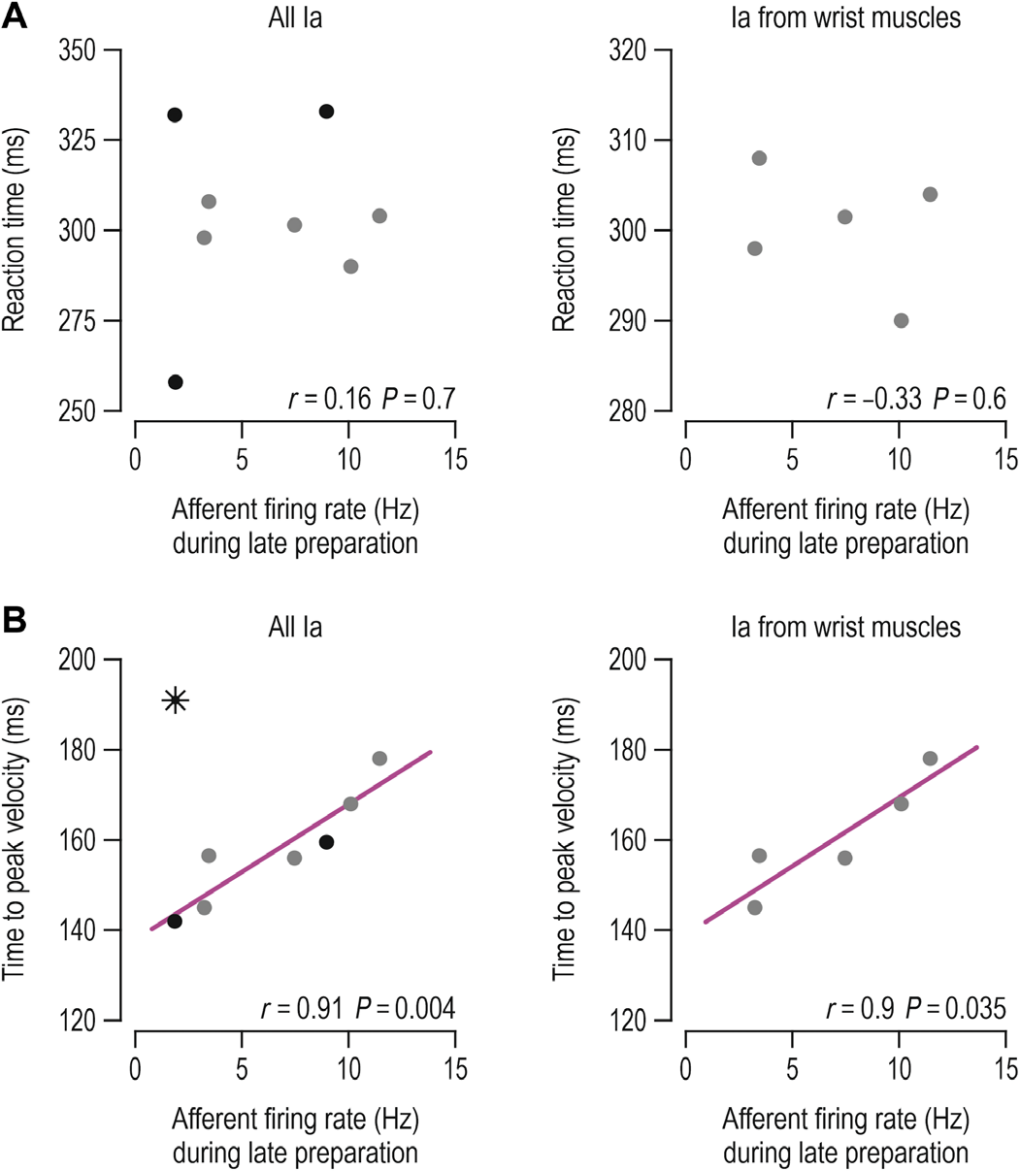
Spindle Ia firing rates at late movement preparation predict performance during reaching. Throughout, each data point represents the average (median) value of a single participant/afferent across trials where reaching the target required stretch of the spindle-bearing muscle. The left column of panels represents all Ia afferents, including those originating from digit extensor muscles (black dots), and the right pertains to Ia from wrist muscles (gray dots). (**A**) Horizontal axes represent firing rates during the late preparation epoch (epoch 3 as defined in Fig. 2B), and vertical axes represent reaction time, i.e., the time between onset of the go cue and onset of the reaching movement. (**B**) Left: Vertical axes represent time between onset of reaching and the point of initial peak velocity during the reaching movement. With the exception of one afferent (black star), there was a strong positive relationship between Ia firing during preparation and time to peak velocity. Right: For the subset of muscles engaged in powering hand movement in the current task, movement performance was well described by the same relationship (i.e., 3-ms delay in attaining peak velocity for every additional spike per second). The relationship between spindle Ia responses at late preparation and subsequent reaching performance can be understood in terms of the spindle’s role in negative feedback circuits (i.e., stretch reflexes).

It is known that movement preparation benefits performance by lowering reaction time (*8*), with a positive relationship existing between preparation delay length and movement quality (*9*). Although we found no relationship between type Ia firing rates observed during late preparation (i.e., epoch 3) and reaction time (Fig. 3A), there was a strong relationship between wrist type Ia responses at epoch 3 and time to peak velocity during reach, with *r* = 0.9 and *P* = 0.035 (Fig. 3B, right). Every unit increase in firing rate during preparation involved an additional 3-ms delay in reaching peak velocity; that is, the regression coefficient was 3. We found no equivalent relationship between this performance measure and kinematic variables (i.e., muscle length and its first and second derivative) or EMG observed at epoch 3 (all *P* > 0.2). The relationship between time to peak velocity and Ia firing at late preparation extended beyond muscles that powered movement in the reaching task. That is, for all but one afferent from digit extensors, the same relationship was found between type Ia firing rates and time to peak velocity (*r* = 0.91, *P* = 0.004, “*b*” coefficient = 0.301; Fig. 3B, left). Note that digit extensors can also affect execution of hand flexion via spinal and transcortical stretch reflex circuits.

### Modulation of stretch reflex gains in reach preparation

**Fig. 4:**
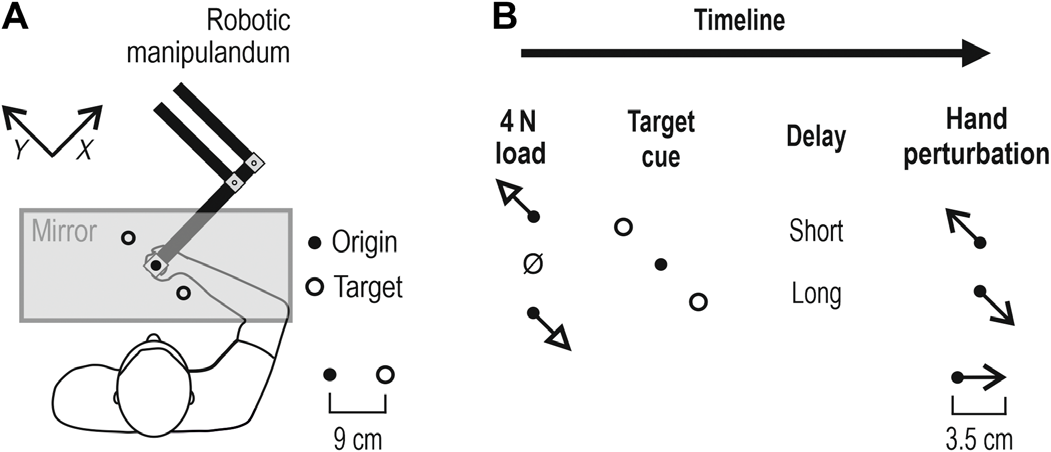
The second experimental setup. (**A**) In experiment 2, participants held the graspable end of a robotic manipulandum. Vision was directed at a one-way mirror, on which the contents of a monitor were projected. Hand position was represented by a visual cursor. Although not shown here, the right forearm rested on an airsled, and the hand was immobile around the wrist (see Materials and Methods for more details). (**B**) Timeline of experimental manipulations. Each trial began by slowly loading the hand to 4 N in the upper left direction (i.e., +*Y* direction) or lower right direction (−*Y* direction), or there was no load (“null” load). The participants had to maintain the hand immobile at origin despite any loading. One of two visual targets (+*Y* or −*Y* direction) was then suddenly cued by turning red, and this state lasted for a relatively short delay (250 ms) or long delay (750 or 1250 ms). These preparatory delays correspond to the middle of epochs 1 to 3 (Fig. 2, B and C). At the end of the delay, the hand was rapidly perturbed toward or in the opposite direction of the cued target. The perturbation lasted for 150 ms; at its end, the go signal was given (cued target turned green), and movement to the target had to be actively completed. Cursor position was frozen during the perturbation. Trials were block-randomized; hence, perturbation direction was unpredictable even after experiencing a particular load, cue, and delay.

**Fig. 5:**
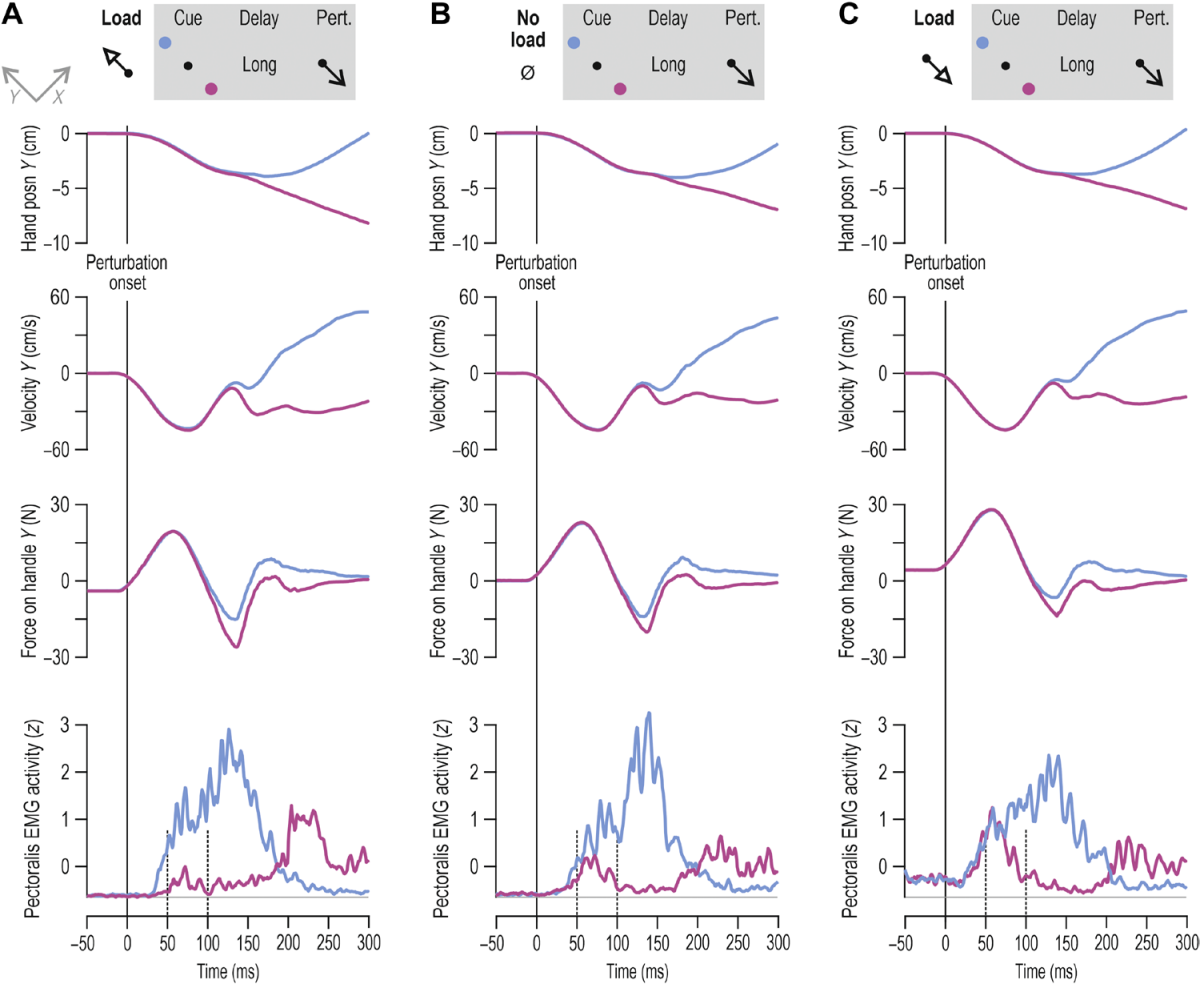
Representative data from a single participant in experiment 2. Relevant median signals from a single participant when perturbations in the −*Y* direction stretched the pectoralis muscle following a 750-ms preparatory delay. The above occurred after first applying a load in the direction of pectoralis shortening (**A**), when there was no external load (**B**), or after first applying a load in the direction of pectoralis stretch, promoting increased pectoralis activity for maintaining the start position (**C**). Throughout, purple traces represent trials where reaching the cued target required pectoralis stretch, and blue traces represent trials where the cued target required pectoralis shortening. Deviations of the hand along the *x* axis were negligible during the perturbation and hence are not plotted in the current figure for clarity. Data are aligned to the onset of the position-controlled haptic displacement (time 0), defined as the point where movement speed reached 5% of initial peak value.

Muscle spindles are known to play a central role in shaping stretch reflex responses. A substantial goal-dependent modulation of spindle gains could lead to equivalent changes in negative feedback gains. We tested this prediction stemming from experiment 1 (i.e., Fig. 2C), by assessing stretch reflex function at the level of the whole upper limb. Namely, in experiment 2, participants performed a version of the instructed-delay reaching task by holding the graspable end of a robotic manipulandum with their right hand (Fig. 4A). The hand could then be mechanically loaded in either the upper left (“+*Y*”) or lower right direction (“−*Y*”), or there could be no load. One of two possible targets would then be cued by turning red (+*Y* or −*Y* direction), and after either a “long” or relatively “short” preparatory delay (see Materials and Methods for more details), the hand would be perturbed in the same or opposite direction as the target (Fig. 4B). Even when perturbations were in the direction of the cued target, participants had to complete the planned movement themselves as the size of the imposed displacement was only about a third of the distance to the target. This ensured that movement control was required on every trial of this task. Figure 5 displays the median responses of a representative participant. Despite identical displacement during the haptic perturbations, visual inspection of the EMG signal from the unloaded pectoralis indicates a clear difference at spinal SLR latencies as a function of cued target (i.e., 25 to 50 ms following perturbation onset; Fig. 5A). This difference is congruent with the afferent findings: a relative suppression of the SLR response when preparing to stretch the pectoralis (purple) rather than shorten it (blue). This suppression disappeared at high background activation levels of the pectoralis, induced by an external load applied before the haptic perturbation (Fig. 5C). A goal-dependent suppression of LLRs (i.e., EMG 75 to 100 ms after perturbation onset) was evident across all load conditions.

**Fig. 6:**
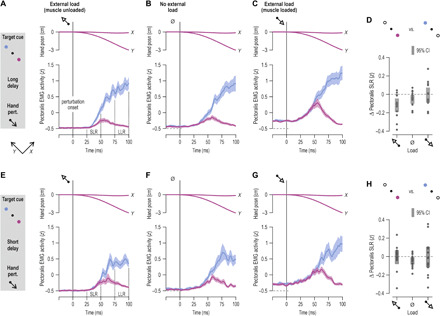
The goal- and delay-dependent modulation of stretch reflex gains is congruent with the preparatory tuning profile of muscle spindles. (**A** to **C**) Mean hand position (posn.) and mean rectified pectoralis EMG activity across participants (*N* = 14) when an external (pre-)load was first applied in the direction of pectoralis shortening (A), when there was no external load (B) (but note increased EMG levels before time 0 due to co-contraction), and when an external load was applied in the direction of pectoralis stretch (C). Shading represents ±1 SEM. Data are aligned to the onset of the haptic perturbation (time 0). As the schematic on the far left indicates, the data represent trials where the preparatory delay was relatively long and the subsequent perturbation stretched the pectoralis. SLR denotes the epoch associated with the spinal stretch reflex and LLR the epoch associated with the long-latency stretch reflex or R3 (for LLR analyses, see Results and Fig. 7). Kinematic data pertaining to the blue condition are also plotted but are obscured. (**D**) Difference in mean pectoralis EMG activity (purple minus blue) in the spinal SLR epoch, corresponding to the data shown in (A) to (C). Dots represent individual participants, and thick vertical lines represent 95% CIs. (**E** to **H**) As top row of panels but representing trials where the preparatory delay was relatively short (0.25 s).

Figure 6 (A to C) represents the equivalent to Fig. 5 for all participants. The same trends can be seen in continuous EMG signals, that is, a goal-dependent suppression of pectoralis SLR and LLR. To concentrate on the effect of cued target while accounting for known effects, such as the universal increase in SLR magnitude that accompanies muscle loading (*12*–*14*), the EMG signals for each muscle, load, and delay condition were contrasted (subtracted) as a function of target cue. This effectively isolated any effect of target cue on SLR responses (but see also LLR analyses across loads). Throughout, we only analyzed EMG signals from stretching muscles (i.e., particular pairs of muscle and perturbation direction) to concentrate on stretch reflex responses. When the preparation delay was long (Fig. 6D), single-sample *t* test indicated a significant suppression of pectoralis SLR when preparing stretch under the “muscle-unloaded” condition, i.e., when an external (pre-)load was applied in the direction of muscle shortening [*t*(13) = −3.5 and *P* = 0.004]. There was also a significant effect of goal on SLR under the no-load condition [*t*(13) = −2.5 and *P* = 0.025], but there was no relative suppression as a function of target cue under the “muscle-loaded” condition, i.e., when the external load was applied in the direction of pectoralis stretch [*t*(13) = −0.23 and *P* = 0.82]. When the preparation delay was relatively short (250 ms; Fig. 6, E to H), there was no suppression of SLRs when an external load was applied in either direction (*P* > 0.8), but there was a weak suppression effect under the no-load condition, with *t*(13) = −2.5 and *P* = 0.025. A congruent pattern of effects was observed for the posterior deltoid muscle (fig. S4). Specifically, when the preparation delay was long, there was a goal-dependent suppression of deltoid SLR under the muscle-unloaded condition [*t*(13) = −3.7 and *P* = 0.002]. There was also significant suppression of SLR under the no-load condition when the delay was short [*t*(13) = −3.3 and *P* = 0.006].

**Fig. 7:**
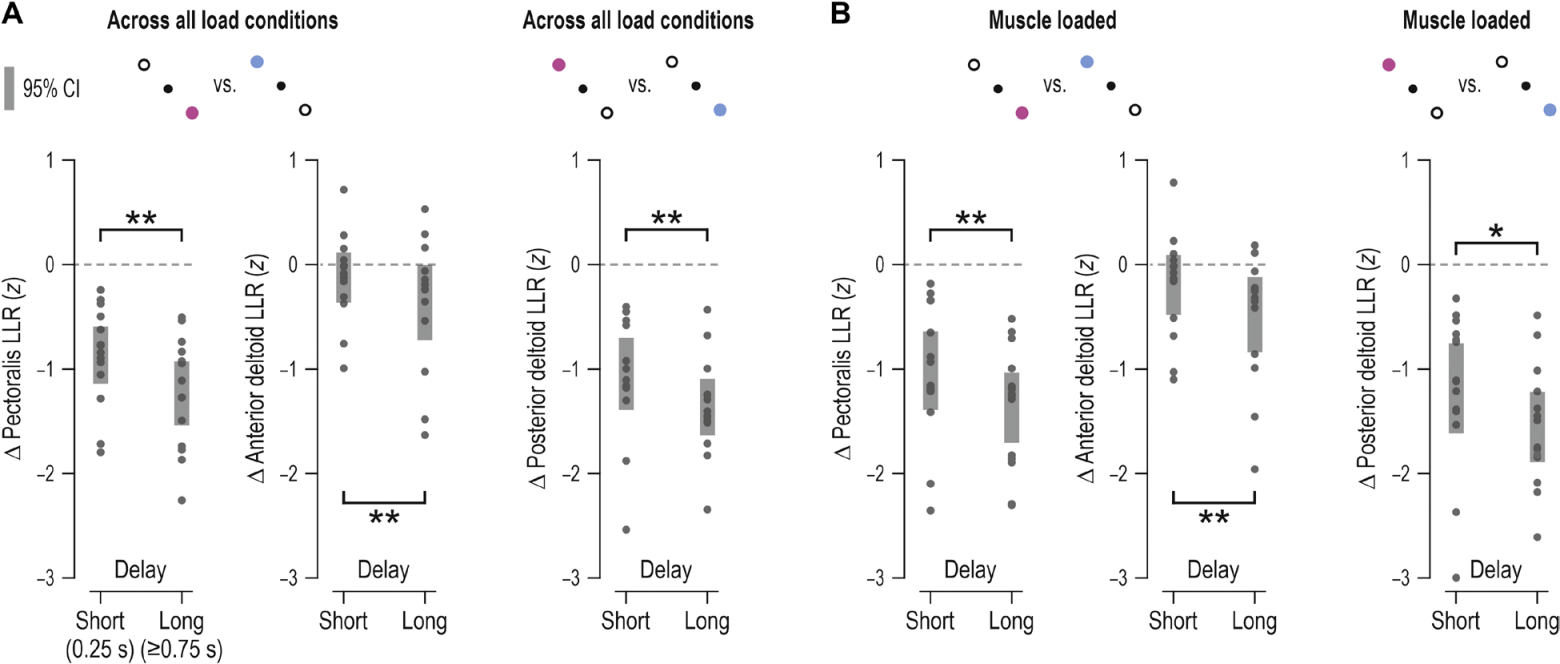
LLR gains reflect the stronger goal-dependent suppression of spindle signals observed at longer preparatory delays. Goal-dependent difference in EMG responses of all recorded shoulder muscles at the LLR epoch (as indicated in Fig. 6A), with regard to the relatively short (250-ms) and long (≥750-ms) preparatory delays used in experiment 2. More negative values indicate stronger goal-appropriate behavior (i.e., relative suppression of stretch reflex gains for muscles that must stretch when reaching the cued target). Throughout, each data point represents the average value of a different participant (*N* = 14), and thick vertical lines represent 95% CIs. Asterisks indicate *P* values following a within-measures *t* test, with double asterisks indicating *P* < 0.01 and single asterisk indicating *P* < 0.05. These results demonstrate a weaker goal-dependent modulation of LLRs when the preparatory delay is short, regardless if contrasted across all load conditions (**A**), or only for the cases where the muscle was externally loaded, i.e., the load was applied in the direction of muscle stretch (**B**). The short delay here was 250 ms, which is substantially longer than the previously reported minimum delay for inducing full expression of goal-dependent LLR responses following perturbations of the upper limb (i.e., 100 to 150 ms). In contrast, the effect of delay length on LLR gains is generally congruent with the temporal evolution of spindle tuning (Fig. 2, B and C).

It is already well established that LLR (or “R3”) responses are goal dependent and influenced by proprioceptive feedback. As can be appreciated by visually inspecting the EMG traces of the pectoralis (Fig. 6) or posterior deltoid (fig. S4), LLR responses were congruent with the goal-dependent afferent results: There is a relative suppression of gains when the target cue is associated with stretch of the particular muscle. However, analyses of LLR responses confirmed an even closer connection to the afferent suppression pattern. Specifically, across all load conditions, there was a stronger goal-dependent suppression of LLR responses following a long rather than a short preparatory delay, with *t*(13) = −3.63 and *P* = 0.003, *t*(13) = −3.45 and *P* = 0.004, and *t*(13) = −3.2 and *P* = 0.007 for the pectoralis, anterior, and posterior deltoid muscle, respectively (Fig. 7A). Our analyses found no significant effect of delay length on LLRs of biceps and triceps muscles (all *P* > 0.05). Performing the same analyses only across cases where the muscles were loaded (i.e., load applied in the direction of muscle stretch) produced equivalent positive findings for shoulder muscles (Fig. 7B) and again no significant effects for “elbow” muscles. However, increasing the workspace of the center-out task in a third experiment (i.e., increasing task demands) not only reproduced the main positive findings of experiment 2 but also revealed an effect of delay length on LLR responses of elbow muscles.

**Fig. 8:**
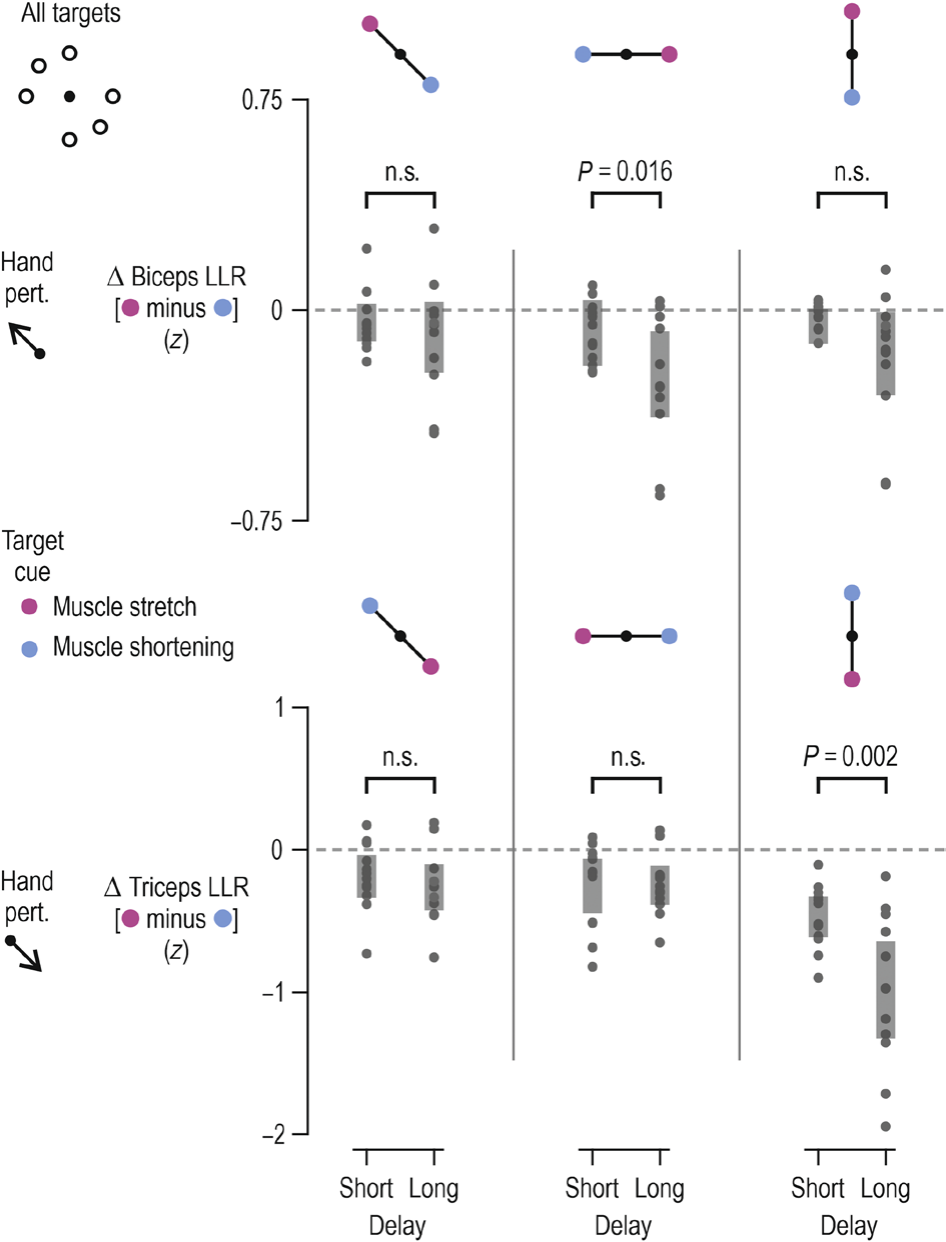
LLR gains of biceps and triceps are also suppressed as a function delay when a larger workspace is involved. As in Fig. 7, but here, the *z*-normalized EMG data originate from experiment 3, where six targets were used (i.e., three axes of motion: vertical, horizontal, and diagonal). The data are collapsed across all load conditions. More negative values indicate stronger goal-appropriate behavior (i.e., relative suppression of stretch reflex gains for muscles that must stretch when reaching the target). Throughout, each data point represents a different participant, and thick vertical lines represent 95% CIs. *P* values resulted from within-measures *t* tests. As the case in experiment 2, the LLR responses of biceps and triceps were not significantly different as a function of delay length when preparing to reach targets along the diagonal axis (left column; only axis used in experiment 2). However, such effects are observed for the biceps brachii and triceps lateralis when preparing to act along the horizontal axis (middle column) and vertical axis (right column), respectively. This suggests that the larger workspace used in experiment 3 (versus 2) induced goal-dependent proprioceptive control of a larger group of muscles, but this control occurred selectively across the task’s dimensions.

Specifically, a third experiment implicating a larger number of visual targets produced equivalent results for pectoralis SLR (i.e., experiment 3; fig. S5). When the preparation delay was relatively long and the pre-load was in the direction of pectoralis shortening (fig. S5, A to C), there was a goal-dependent suppression of SLR gains, with *t*(11) = −4.1 and *P* = 0.002 (fig. S5D). Although for most participants, SLRs were suppressed under the no-load condition as well (middle column in fig. S5D), the overall difference was deemed not significant (*P* = 0.2; note the one deviant value of >0). There was also a small but significant suppression of SLRs under the no-load condition when the delay was short (fig. S5, E to H), with *t*(11) = −2.8 and *P* = 0.017. As in experiment 2, LLRs of shoulder muscles reflected the spindle suppression pattern. That is, across all load conditions, goal-appropriate suppression of shoulder muscle LLR was stronger if a long rather than short delay preceded congruent perturbations, with *t*(11) = −2.42 and *P* = 0.034, *t*(11) = −2.22 and *P* = 0.048, and *t*(11) = −2.3 and *P* = 0.042 for the pectoralis, anterior, and posterior deltoid muscle, respectively. A significant effect of delay length was also found for the triceps lateralis, with *t*(11) = −3.74 and *P* = 0.003. To better contrast the seemingly conflicting results of experiments 2 and 3 regarding the effect of preparatory delay length on elbow muscles, the data were contrasted separately for each of the three main axes of motion involved in experiment 3: diagonal (as in experiment 2), vertical, and horizontal (Fig. 8). As in experiment 2, there was no effect of delay length on biceps and triceps LLR responses when action was required along the diagonal axis (*P* > 0.05). However, along the horizontal dimension, there was a stronger goal-dependent suppression of biceps LLR following a long delay [*t*(11) = −2.73 and *P* = 0.016], and the same effect was evident for the triceps when target cues required action along the vertical axis [*t*(11) = −4.02 and *P* = 0.002). The above results suggest that the increased demands (i.e., larger workspace) of experiment 3 necessitated the proprioceptive control of a larger group of muscles in a task-dependent manner (Fig. 8), even though the perturbations themselves where always applied along the diagonal axis, as in experiment 2.

## DISCUSSION

Our results demonstrate that movement preparation involves goal-directed tuning of muscle spindle receptors and stretch reflex gains. We therefore suggest that one function of central preparatory activity is to adjust muscle mechanical compliance according to task goals. Our findings are congruent with classic results concerning preparatory activity in the central nervous system (CNS) and its two hallmarks, which are (i) that preparatory activity should not overtly affect concurrent muscle force and (ii) preparatory activity needs to somehow facilitate the planned voluntary movement. The current study helps bridge the gap between traditional views where preparatory activity is seen as representing specific movement parameters (*2*, *4*–*6*) and the more recent claims that movement preparation shapes an initial state of a dynamical system whose evolution produces the planned movement (*7*). We show that such an “initial” state may partly pertain to the state of the peripheral proprioceptive apparatus, which can predispose the system for goal-directed reflex responses from muscles whose level of compliance can substantially affect execution of the planned task (e.g., Figs. 6 to 8). This preparatory mechanism may represent a significant source of individual differences in motor performance. We found that higher levels of tonic type Ia discharge at late preparation are associated with worse reaching performance (i.e., larger delays in attaining peak velocity). This relationship can be understood in terms of the spindle’s role in generating negative feedback via stretch “reflexes.”

The current study is the first to record muscle afferent responses during movement preparation (i.e., over a dedicated delay period) in a context where voluntary reaching movements were actually made. One other study (*15*) implicating the lower limb looked at spindle responses when anticipating the need to make a contraction that would oppose an expected external perturbation. No preparatory effects were found in the aforementioned study, but we believe our paradigm better reflects the state of affairs when reaching in everyday life, as the task combined true reaching intention and action. There has also been strong evidence of preparatory activity in spinal interneurons (*16*), but our study is the first to document preparatory changes in sensory elements of the peripheral nervous system. Hence, the results show that preparatory activity can also be associated with implementation of a movement plan (i.e., application of “control policy”), an issue that has been unclear up to this point (*17*).

It is well known that there are no goal-dependent changes in skeletal muscle state during movement preparation, despite vigorous changes in CNS preparatory activity as a function of goal (*1*–*3*, *7*, *17*). Accordingly, there were no systematic deviations in muscle kinematic and surface EMG signals during movement preparation in our study. This suggests that the observed goal-dependent changes in type Ia firing (and the equivalent modulation of SLR and LLR gains) were due to independent fusimotor control of muscle spindle receptors. All recorded type Ia afferents exhibited a goal-dependent decrease in their firing rates (Fig. 2C), whereas no consistent modulation was observed in type II afferents. The observed change in spindle firing is compatible with a decrease in dynamic γ motor neuron drive to muscles preparing to stretch. These “dynamic” fusimotor neurons only affect primary spindle receptors (i.e., type Ia responses), and a substantial decrease in dynamic fusimotor drive is known to induce some decrease in background (tonic) Ia firing (*18*). However, dynamic fusimotor supply has a stronger positive effect on the gain of the primary spindle to dynamic muscle stretch (*11*, *18*). The involvement of dynamic fusimotor drive is not only supported by the lack of preparatory changes in type II firing, although it has been shown that a small number of spindle afferents can provide a reliable representation of the underlying population responses [e.g., (*18*)]. An alternative interpretation could implicate goal-dependent changes in “static” fusimotor supply. However, static fusimotor activity has a negative effect on primary spindle gain; that is, a decrease in static fusimotor drive would likely entail an increase in the spindles’ dynamic response to stretch. This is contrary to the relative suppression of stretch reflex gains when preparing stretch of the perturbed muscle (e.g., Fig. 6A and fig. S5A).

We also found an increase in Golgi afferent (“type Ib”) firing when preparing either muscle shortening or stretch. It is theoretically possible that the increase in Ib firing across all targets represented some small anticipatory co-contraction that was not detected by surface EMG (hence likely to only involve a small subset of muscle fibers). Nevertheless, in this scenario, the observed decrease in spindle Ia firing when preparing stretch is thought to be due to very small (and externally undetectable) concurrent muscle shortening. However, the lack of a corresponding decrease in Ia firing when preparing muscle shortening infers a goal-dependent increase in dynamic fusimotor drive under this condition. Note that muscle spindle gains are not necessarily affected by background mechanical loading. When imposing stretch of the isometrically loaded radial wrist extensor, no clear net difference in spindle sensitivity is found, as an approximately equal number of dynamic and static fusimotor effects appear, with these two having opposite effects on spindle gain (*19*). Overall, our spindle afferent and stretch reflex findings are compatible with a preparatory goal-directed change in γ fusimotor drive to task-relevant muscles. Determining the central origin of fusimotor control was outside the scope of the current study. Identifying the origin or specific descending pathways associated with preparatory fusimotor control would help elucidate further the underlying mechanisms. However, we believe that sufficient evidence supports the main novel claim of this study, i.e., that there is advantageous preparatory tuning of muscle spindle receptors.

Up to this point, the general expectation of no specific role for spindle receptors in movement preparation has been formulated indirectly, primarily through behavioral studies examining spinal SLR responses in surface EMG from the upper limb. Although there has been some evidence of goal-dependent modulation of SLRs, both at the level of digits (*20*) and at more proximal areas (*21*), previous studies have not identified goal-dependent spinal SLR responses. However, such goal-dependent responses are consistently found at transcortical latencies (*22*). The results of experiments 2 and 3 suggest that, given a particular experimental design, goal-dependent modulation of reflex responses can be consistently unmasked. Two important elements of the adopted experimental design are the systematic manipulation of background load and ensuring that movement control is required for reaching every target (including in trials where perturbations displaced the hand toward the cued target). With regard to the latter element, the intent to actively engage in attaining a movement’s goal may be necessary for invoking preparatory proprioceptive control. Regarding the former, many previous studies either did not account for the background activation levels of muscles or deliberately preloaded muscles to ensure detectable levels of surface EMG in the SLR epoch. We show that strongly loading a muscle can potentially obscure evidence of goal-dependent proprioceptive tuning (e.g., Fig. 6, A to C). That is, our results show that load-related or “automatic” gain-scaling (*12*, *13*) of SLRs for the purposes of postural control may compete or otherwise interfere with target-dependent tuning of spinal SLRs.

It is known that LLR responses are robustly goal dependent and largely immune to load-based gain-scaling. Accordingly, across all load conditions, we show a modulation in LLR gains that closely reflects the spindle’s preparatory tuning profile (e.g., Figs. 6 and 7). That is, we show a goal-appropriate modulation of gains that is stronger following long than relatively short preparatory delays. Crucially, the short delays used in our study are considerably longer than the minimum time required for shaping transcortical reflex responses via selective CNS processing of proprioceptive signals (*23*, *24*). The current LLR results are supportive of an independent and relatively slow-evolving mechanism acting on proprioceptors during preparation for active reaching. It is known that dynamic fusimotor neurons innervate very slow “bag1” intrafusal muscle fibers. Effects on spindle firing take several hundred milliseconds to completely disappear following immediate cessation of artificially induced dynamic fusimotor stimulation, e.g., (*25*). Our findings are also compatible with reported improvements in reach movement quality that occur for preparation delays of >150 ms (*9*).

The current findings highlight that muscle spindle receptors and their independent motor system can serve more decisive and task-dependent roles in sensorimotor control than generally thought. Traditionally, the spindle organ has been seen as a peripheral mechanoreceptor that provides reliable information about a muscle’s kinematic state. An interesting recent proposition is that the mechanoreceptive part of spindles responds best to force-related rather than length-related variables, as shown in passive (“electrically quiescent”) muscles (*26*). When performing continuous active sinusoidal movements with a single digit in the presence of external loads, we have also shown that spindle afferent activity from digit extensors best encodes a combination of velocity and net joint torque (*27*). However, our more recent work examining spindle responses in visuomotor learning (i.e., visuomotor rotation) revealed fundamental changes in spindle output as a function of task stage (e.g., encoding position only in the “washout” stage), with no fundamental differences in kinematic state across the task’s stages (*28*). Besides indicating that the fusimotor system is a specific contributor in visuomotor learning, the aforementioned study showed that spindle output can be modified on the basis of changes in the visual environment alone. This is in line with the findings of the current study (Figs. 1 and 2). Very recent spindle afferent recordings during passive movement of the foot also indicate that visual feedback can affect spindle output (*29*). Accumulating evidence therefore suggests that human spindles can transcend their traditionally ascribed role as mechanoreceptors invariably encoding some muscle state regardless of context or goal. In cats, it has been shown that spindles can receive a different “fusimotor set” (*30*) depending on the behavior the animal is engaged in, but the specific benefit of the different dynamic fusimotor sets has been unclear, and these sets generally seem to reflect the alertness state of the animal. Here, we demonstrate spindle gain modulation as a function of visually determined goals within the same behavior (reaching), including evidence of how this spindle tuning can promote motor performance.

The traditional view of spindles as basic mechanoreceptors is the one currently adopted by prevalent computational frameworks of sensorimotor control (*31*, *32*). Part of these suggest that our brain predicts the sensory consequences of action and then compares internal predictions and actual incoming sensory signals, with no discrepancy between the two indicating agency of action. With regard to primary muscle spindles in the context of planned reaching movements, our results suggest that the nervous system does more than these computational frameworks describe. Presumably still based on internal models and predictions of future outcomes given an intention or goal (*31*, *32*), the system seems able to proactively choose and implement a change in sensory feedback gains at source (Fig. 2, B and C). That is, in planned voluntary reach, the “controller” can proactively modify the controlled body part or “plant” (i.e., adjust sensitivity of the plant’s sensors) to facilitate the intended action, such as by preventing consequences (negative feedback) that would otherwise interfere with execution of the action. Beyond its role in planned reaching, the independent and direct control of sensors via γ motor neurons may well constitute an important overarching third dimension in sensorimotor control, in addition to (i) top-down processes leading to α motor neuron control and (ii) the selective gating and internal processing of sensory signals. By demonstrating advantageous tuning of spindles in movement preparation, the current study supports the notion of a “third way” in which the nervous system can exert goal-directed sensorimotor control.

## MATERIALS AND METHODS

### Experimental design

#### Microneurography platform

The participants were seated reclined on an adjustable chair with their right forearm resting on a cushion. The activity in single afferents from wrist or digit actuator muscles was recorded along with wrist joint kinematics and EMG activity from relevant forearm muscles (Fig. 1A). Participants used their right hand to perform a classic center-out reaching task, where each target is first cued before a go cue to move is issued (the task is described in more detail below). A clamp proximal to the wrist stabilized the upper arm and helped prevent electrode dislocations, but hand movements about the wrist were fully unrestrained in this setup. In “classic” center-out reaching tasks, target location is normally presented on a monitor and so is the visual feedback on the location of the hand, represented by a moving cursor. The approach was the same here: Visual feedback was provided by a monitor that was placed across from the participants and elevated at about their eye level. They controlled the two-dimensional (2D) location of a cursor on the monitor through wrist movements recorded by a FASTRAK sensor attached to the dorsal surface of the hand with double-sided tape. The initial posture of the hand represented a neutral wrist position, which, in turn, corresponded to the “origin” position of the cursor (Fig. 1A). In this neutral position, the hand (e.g., third metacarpal joint) was aligned with the long axis of the forearm, and to hold this position against gravity, the participants had to produce a constant low-level contraction mainly in the extensor carpi radialis. Wrist radial/ulnar rotations controlled cursor movements in the vertical visual axis, and flexion/extension controlled cursor movements in the horizontal axis. One degree movement at the wrist corresponded to 0.7-cm on-screen movement of the visual cursor. Visual targets not involved in an ongoing trial were represented as light brown circle outlines (1.5 cm radius; origin outline had 1 cm radius). The targets were placed symmetrically around the origin in 45° intervals so that movements in all major directions were induced (Fig. 1A). The distance between the center of the origin and the center of a target was 12°, but a minimum wrist movement of 10° was required for successfully reaching from origin to target (i.e., edge to edge).

#### Robotic platform

Here, the participants were seated upright on an adjustable chair, and their right hand grasped the handle of a robotic manipulandum (KINARM end-point robot, BKIN Technologies, CA; Fig. 4A). Although not displayed in Fig. 4A, the participant’s right forearm was placed inside a thin cushioning foam structure attached to a custom-made airsled; this structure supported the participant’s forearm and allowed frictionless movement of the arm in a 2D plane. A piece of leather fabric with Velcro attachments was wrapped tightly around the forearm and hand, reinforcing the mechanical connection between the airsled, the handle, and the hand. This attachment also fixated the hand so it remained immobile about the wrist and straight (i.e., aligned with the forearm) throughout the experiment. The forces exerted by the participant’s right hand were measured by a six-axis force transducer (Mini40-R, ATI Industrial Automation) embedded in the handle, and the system also generated kinematic data with regard to the position of the handle. The KINARM also produced controlled forces on the hand, both for the background (pre-)loading of muscles and for creating position-controlled mechanical perturbations. Surface EMG was concurrently recorded from seven muscles actuating the right arm (see the relevant section for more details). Visual feedback was very similar to that presented in the microneurography experiment, but in the robotic platform, visual stimuli were displayed in the plane of movement by way of a one-way mirror, on which the contents of a monitor were projected. The participants had no direct vision of their hand (Fig. 4A), but position of the hand was visually represented by a white dot (“cursor”; 1 cm diameter). Targets not involved in an ongoing trial were displayed as circle outlines (1.2 cm radius; origin outline had 0.65 cm radius). The targets were placed symmetrically at a distance of 9 cm from the origin.

#### Microneurography—Hand movement task

In the behavioral task associated with microneurography, the participants (*n* = 9) were instructed to place the cursor inside the origin circle and wait there immobile before a trial could start. After a random wait period (0.5 to 2.5 s), one of the eight different targets would suddenly turn from a circle outline to a filled red circle of the same size. This indicated which target the participant had to reach once the go cue appeared. The presentation of targets was block-randomized. The go cue in this case was the red target suddenly turning into a green outline of the same size. For the majority of the participants (seven of nine), the time between onset of the target cue (red circle) and onset of the go cue was a fixed 1.5 s (“preparatory period”). To assess whether any major afferent firing patterns during movement preparation were critically sensitive to major characteristics of the particular preparatory period, we used 1 s as the preparatory period with one participant and 1.5 s + random time (1 to 500 ms) for another. No substantial differences in firing patterns were found between these afferents and the rest Ia. To aid subsequent analyses, data from the initial 1.5 s were used in the latter case, and in the former case, the data during the 1-s periods were resampled offline to 1.5 s. In all experiments, the participants were instructed to initiate the reach movement promptly upon onset of the go cue and to move at a naturalistic speed. To promote this behavior, participants received visual feedback on their performance upon reaching a target. That is, they received the message “good” if they managed to reach the target within 1 s following onset of the go cue and “fail” if they took longer. After receiving feedback, the participants returned to the origin to initiate the next trial. The task continued until the afferent recording was lost due to an accidental dislocation of the electrode, an all too common occurrence when recording during naturalistically fast active movement (but at least 24 trials, i.e., three blocks of trials, were recorded with each afferent; see below for more details). Trials where movement was initiated prematurely (i.e., before the go cue) were excluded from analyses, but these represented just one trial per afferent on average and in no case more than two trials per recorded afferent. To familiarize the participants with the center-out task and promote good performance at it during microneurography, they practiced the task for ~10 min before microneurography began.

#### Robotic platform—Arm movement tasks

Two experiments were conducted using a robotic platform (experiments 2 and 3), with each experiment using a different set of participants. Before the main task of either experiment, each participant initially performed a brief unperturbed center-out reaching task that was very similar to that during microneurography. This introductory task was included to establish a closer link between the behavioral task in microneurography and the main task applied with the robotic platform (described below). Specifically, in this brief center-out task, participants were instructed to bring the hand in the origin circle and remain there immobile. After a wait period of 1 s + random time (1 to 500 ms), one of the eight peripheral targets/outlines turned into a filled red circle of the same size, indicating which target the participant had to reach once the go cue appeared (go, target turning green). The preparatory period here was a fixed 1.5 s to match the case during microneurography. Participants had to move at a naturalistic speed, and upon reaching a target, they received visual feedback on their performance. Counting from the onset of the go cue, the feedback was “too slow” if the reach movement lasted >1400 ms, “too fast” if <400 ms, and “correct” if the movement duration was in between the two stated extremes. After receiving feedback, the participants returned to the origin to initiate the next trial. There were 80 trials in total (i.e., 10 repetitions × 8 targets), presented in a block-randomized manner, with one set of eight different targets representing a “block.” The task lasted ~5 min.

Following a short break of a few minutes, the participants then performed the main behavioral task. In experiment 2 (e.g., Fig. 6), the main task lasted for ~1 hour, whereas in experiment 3 (e.g., fig. S5), the task lasted ~1.5 hours. The main task was designed to emphasize reflex responses from shoulder actuators, allowing the possibility to extend positive findings to the most proximal areas of the upper limb, although elbow muscle reflexes were also stimulated. Specifically, visual feedback in the main behavioral task of experiment 2 was the same as in the brief introductory task described above, except that two rather than eight targets were used and the cursor position was frozen for the duration of haptic perturbations. Before each trial begun, the participants brought the hand (i.e., cursor) inside the origin circle. After a wait of 1 s + random time (1 to 500 ms), the robotic arm was programmed to elicit a slow-rising 4-N load (rise time of 800 ms, 1200 ms of hold time) in the front and left direction (+*Y* direction) or right and back direction (−*Y* direction), or no load was applied. A substantial load could therefore be present at this point in each trial, with the function of pushing toward one or the other target (Fig. 4). Because the participants were instructed to maintain their hand in the middle of the origin circle during this phase of the trial, the ultimate purpose of this maneuver was loading/unloading of the recorded muscles, primarily the posterior deltoid or pectoralis and anterior deltoid. After an additional 1.2 s where the full force of the load was countered while the hand remained still, one of the targets was cued by becoming a red filled circle. After a preparatory period of either 0.25, 0.75, or 1.25 s, a position-controlled perturbation of the hand occurred (3.5 cm displacement, 150 ms of rise time, no hold period), swiftly moving the hand in the +*Y* or −*Y* direction. The specific preparatory delays were chosen to match the middle of epochs “1 to 3”, as identified in Fig. 2 (B and C). The haptic perturbations were designed to induce the kinematics of a fast naturalistic point-to-point movement (i.e., approximate bell-shaped velocity profile; e.g., see Fig. 5). The robot was allowed to use maximum available stiffness (~40,000 N/m), if necessary, to achieve the desired kinematics on every trial. The KINARM robot was able to reliably impose the required hand kinematics during these perturbations regardless of background load/force conditions. When the haptic perturbation ended (i.e., 150 ms after perturbation onset), the go cue suddenly appeared, and the participants swiftly reached to this highlighted target. The trial ended when the participants kept their hand immobile inside the target for 0.3 s, after which they received visual feedback on their performance (i.e., correct, too fast, or too slow), as per the brief introductory task. The participants then returned their hand to the origin to initiate the next trial. Each block of trials represented 1 repetition of each level of each condition (i.e., block = 36 trials: 2 targets × 2 perturbation directions × 3 preparatory periods × 3 load conditions), and there were 15 repetitions of the complete trial block; that is, the total number of trials was 540. The trials were presented in a block-randomized manner, and therefore, all perturbations were unpredictable to the participants in terms of their timing (onset) and, ultimately, in terms of their direction. The participants had the opportunity to take a short break at the end of each block of trials. “Experiment 3” (e.g., fig. S5) was essentially the same as “experiment 2” except that six targets were used rather than two, and the two preparatory delays were 0.2 and 1.2 s, also referred to as short and long. Each block of trials represented 1 repetition of each level of each condition (i.e., block = 72 trials: 6 targets × 2 perturbation directions × 2 preparatory periods × 3 load conditions), and there were 10 repetitions of the complete trial block; that is, the total number of trials was 720.

### Muscle afferent recordings

Single spikes in afferents originating from either the radial wrist extensor (extensor carpi radialis), the ulna wrist extensor (extensor carpi ulnaris), or the common digit extensor (extensor digitorum communis) were obtained using the technique of microneurography (*33*). The radial nerve of the right arm was targeted, and isolated single action potentials were categorized as originating from spindle or Golgi tendon organ afferents following standard procedures described in detail elsewhere (*27*, *34*, *35*). These procedures included examining afferent responses to ramp and hold stretches of the relaxed spindle-bearing muscle, assessing the variability of discharge, and looking at the nature of responses to isometric contraction and sudden relaxation. In total, 12 muscle spindle afferents (8 “type Ia” and 4 “type II”) and three Golgi tendon organ afferents were recorded from nine participants (minimum of one recorded afferent per included participant). With all afferents, a minimum of 24 movement trials were recorded (i.e., three repetitions of a movement direction), and with some, the recording lasted longer, allowing for more repetitions to be sampled.

As expected, the primary spindle afferents responded with higher overall firing rates to dynamic muscle stretch than muscle shortening. Just one afferent from a digit extensor was not responsive to one of the three stretch target directions (i.e., upper left direction) but was very responsive to the other two stretch directions. Likely causes for such variability include the particular set of fusimotor supply and the precise location of the spindle organ inside the muscle. The number of afferents recorded in this study reflects that in previous studies examining single afferent activity during active movement [e.g., (*27*, *28*, *36*)]. Moreover, it has been shown that a small number of spindle afferents can provide a reliable representation of the firing patterns observed in the underlying afferent population [e.g., (*18*)]. This is not unexpected, as all muscle spindle organs are placed mechanically “in parallel” with the skeletal muscle fibers, and the spindle acts as an integrator of activity from multiple fusimotor fibers.

### Muscle EMG recordings

In the microneurography experiment, custom-build surface electrodes (∅ 2 mm; 12-mm apart) were used for recording EMG from the common digit extensor and digit flexor muscles, as well as from the four main wrist actuators (extensor carpi radialis, extensor carpi ulnaris, flexor carpi radialis, and flexor carpi ulnaris). The location of each electrode on the forearm was chosen using a handheld stimulator probe and isometric contraction/relaxation maneuvers. In experiments 2 and 3, the Delsys Bagnoli system (DE-2.1–single differential electrodes) was used to record surface EMG from the pectoralis, posterior deltoid, and anterior deltoid. We also recorded EMG from the brachioradialis, biceps, and triceps areas. In all experiments, EMG electrodes were coated with conducive gel and attached to the skin using double-sided tape.

### Participants

We recorded afferent activity from 9 adults in the first experiment (mean age of 27 and SD = 3 years; 5 were male), 14 individuals took part in the second experiment using a robotic manipulandum platform (mean age of 24.5 and SD = 4 years; 6 were male), and an additional 12 adults participated in the third experiment using the same platform (mean age of 25 and SD = 5 years; 5 were female). All participants reported having no motor or cognitive disabilities, had normal or corrected vision, gave their written consent before taking part, and were financially compensated. The current experiments were part of research programs approved by the Regional Ethics Committee of Umeå and followed the Declaration of Helsinki regarding research with humans.

### Data sampling and processing

The data generated during the microneurography experiment were sampled digitally using SC/ZOOM. Single action potentials were identified semiautomatically under visual control. The EMG channels recorded during microneurography were root mean square–processed with a rise-time constant of 1.0 ms and a decay-time constant of 3.0 ms; they were then digitally sampled at 1600 Hz. The EMG channels were high-pass–filtered with a fifth-order, zero-phase-lag Butterworth filter with a 30-Hz cutoff. Kinematic and force data from the KINARM platform were sampled at 1 kHz. The recorded EMG signals were band-pass–filtered online through the Delsys EMG system (20 to 450 Hz) and sampled at 2 kHz. These EMG data were also high-pass–filtered with a fifth-order, zero-phase-lag Butterworth filter with a 30-Hz cutoff and then rectified. To be able to compare and combine EMG and afferent data across muscles and participants, the raw data were normalized (*z*-transformed), similar to the procedure described elsewhere (*21*, *27*, *28*). Briefly, for each individual muscle (or individual afferent), all relevant raw data traces were concatenated, and a grand mean and SD were generated. These two numbers were then used to produce the normalized “raw” EMG data for each muscle or produce the normalized firing rate of each afferent (i.e., by subtracting the grand mean and then dividing by the SD). Exemplary untreated raw data are also presented (Fig. 1, B and C). For plotting purposes alone, continuous firing rate signals were smoothed using a 10-ms moving window (i.e., Fig. 2B), and a 5-ms moving window was used for EMG signals (e.g., Fig. 5). Throughout, data tabulations were performed using MATLAB (MathWorks, Natick, MA, USA).

### Statistical analysis

The main statistical approach involved conducting repeated-measures *t* tests and ANOVA and complementary planned comparisons on kinematic, EMG, and normalized spindle firing rate data observed during the preparatory periods (experiment 1) and single-sample *t* tests on EMG data pertaining to spinal and long-latency stretch reflex responses elicited during haptic perturbations (experiments 2 and 3). Specifically, with regard to the analysis of the afferent data, it is known that kinematic variables such as position (i.e., muscle length) and its derivatives as well as spindle-bearing EMG activity can affect spindle output, with muscle velocity (i.e., first derivative of muscle length) believed to normally exert the largest influence. To generate estimates of muscle length (tendon excursion) from the recorded wrist angular data, we used established physiological models (*37*, *38*) as done previously elsewhere (*28*, *34*, *35*). As expected, kinematic and EMG variables represented very small levels of variability during the main period of interest (i.e., immobile hand during the preparatory period; fig. S1). The main analyses of data from “experiment 1” examined potential effects of the goal/target of each trial (i.e., prospective movement direction: muscle stretch versus shortening) during movement preparation, and no systematic variation in kinematic variables or EMG was found as a function of goal (fig. S2).

To investigate the impact of goal, we grouped different trials into those associated with clear stretch versus clear shortening of the spindle-bearing muscle (Fig. 2A) based on the aforementioned physiological models, but this grouping is nevertheless intuitive and straightforward (e.g., for the radial wrist extensor, targets requiring wrist flexion and/or ulna deviation were classified as “muscle stretch” targets). For each single afferent, the normalized raw data across trials were first aligned to the onset of the target cue. To more clearly isolate possible changes in firing rate as a function of target, the median firing rate observed during the 0.5-s period before target onset (“baseline”) was subtracted from the entire firing rate signal on a trial-by-trial basis. The firing rate signals were collapsed across trials to get a single averaged (median) response signal for each afferent and target group (i.e., stretch versus muscle shortening targets). Averaging across all afferent signals for each target group gave an estimate of population responses (Fig. 2B). From each averaged afferent signal, the data points used in statistical tests (ANOVA/*t* test) were the median value across each of three epochs of equal length, termed epochs 1, 2, and 3 (Fig. 2, B and C). The data points pertaining to individual spindle afferents (i.e., Fig. 2C) were entered into a two-way repeated-measures ANOVA, of the design 2 (goal/direction) × 3 (epoch). Single-sample *t* tests, planned comparisons, and simple linear correlations were also performed. The same single-sample *t* test analyses were also performed with kinematic and EMG data, as described in Results. For reference, across the eight Ia afferents (Fig. 2, B and C), the median empirical firing rate during the baseline period was 8.3 spikes/s, and firing rates decreased by an average (median) of 30% in epoch 3, compared to baseline.

With regard to stretch reflex responses to haptic perturbations (i.e., experiments 2 and 3), the analyses focused on established time periods known to reflect the output of spinal and supraspinal stretch reflex circuits. Specifically, across all experiments, the onset of movement or kinematic perturbation was defined as the point where movement velocity (i.e., first derivative of Euclidean displacement) exceeded 5% of peak velocity during the perturbation phase (note that the position-controlled perturbations had an approximate bell-shaped velocity profile). Using the onset of the kinematic perturbation to signify time zero, the spinal stretch reflex response (SLR) is defined as that occurring in the epoch 25 to 50 ms after perturbation onset, whereas the LLR response (aka R3) is defined as that occurring in the epoch 75 to 100 ms after perturbation onset, e.g., (*39*, *40*). The magnitude of the SLR and LLR response was representative of changes in gain, as the same input (perturbation) was provided when the hand was at a common start position. An epoch of the same length as the SR one was used for representing preperturbation muscle activity (i.e., −25 to 0 ms). Unlike the case of the behavioral task during microneurography, the participants received no prior training in the main behavioral task with the robot. As the situation of interacting with a robot that perturbs one’s hand on every trial is also less than completely naturalistic, the initial five repetitions of each trial type were considered to be “familiarity” trials and were excluded from analyses; excluding a number of initial trials is a common approach in similar robot-based sensorimotor control studies. In experiment 2, three preparatory delays were used (0.25, 0.75, and 1.25 s), reflecting the middle of each of the three epochs used for analyses in experiment 1 (Fig. 2B). As expected from the afferent findings (Fig. 2C), visual inspection on EMG signals confirmed that a similar suppression of spinal SLR occurred for the two longer delays (e.g., Fig. 5 represents trials where the delay was 0.75 s). The data were therefore collapsed across the two delays to represent one long delay condition (Fig. 6). The relevant data used in statistical analyses for each participant were generated by first creating averages (medians) of EMG signals across repetitions of a relevant trial type that involved stretch of the particular muscle (i.e., EMG signals during muscle shortening were not analyzed in the current study as we were interested in stretch reflex responses). The average value within the epoch of interest was then taken, producing a single data point per muscle and trial type. To simplify analyses (i.e., concentrate on the main manipulation of interest while accounting for known effects of, e.g., muscle loading), for each individual muscle, EMG data of a particular load and/or delay were contrasted in terms of the target goal, generating a single data point that was ultimately used for statistical analyses as part of a single-sample *t* test (see, e.g., Fig. 6, D and H).

All statistical comparisons were two-tailed, and the overall baseline statistical significance level was 0.05. Tukey’s post hoc test was used for any post hoc analyses. No statistical methods were used for predetermining sample sizes, but the sizes used are similar to those reported in previous studies. Data normality was confirmed using Shapiro-Wilk test for samples with <50 data points and Lilliefors test for larger samples. Statistical tests were performed using either matlab (MathWorks, Natick, MA, USA) or statistica (StatSoft Inc., USA).

## SUPPLEMENTARY MATERIALS

Supplementary material for this article is available at http://advances.sciencemag.org/cgi/content/full/7/9/eabe0401/DC1

## Appendix

View/request a protocol for this paper from *Bio-protocol*.
